# The Bacteriological Diagnosis of Plague

**Published:** 1901-06-08

**Authors:** 


### THE BACTERIOLOGICAL DIAGNOSIS OF PLAGUE.

AT the last meeting of the Epidemiological Society Dr. Klein pointed out that although there was little difficulty in the diagnosis of plague during an epidemic, or on board vessels coming direct from infected ports, there were under other circumstances cases in which buboes were the only symptoms, and which could not be diagnosed with any certainty except by means of a bacteriological examination of the exudation or pus in the glands. The bacilli, he said, teemed in the pulmonary exudation and expectoration of the pneumonic, and in the blood of the septicæmic and hæmorrhagic types, but in the bubonic they were practically limited to the spleen and the implicated glands. This explained the high infectivity of the former and the rarity with which the latter was communicated until after the rupture of the abscess.

